# Population connectivity: dam migration mitigations and contemporary site fidelity in arctic char

**DOI:** 10.1186/1471-2148-11-207

**Published:** 2011-07-14

**Authors:** Jens Wollebæk, Jan Heggenes, Knut H Røed

**Affiliations:** 1 The Norwegian School of Veterinary Science, Dep. of Basic Sciences and Aquatic Medicine, Box 8146, Dep. 0033 Oslo, Norway; 2 Telemark University College, Dep. of Environmental Sciences, Hallvard Eikas Plass, 3800 Bø i Telemark, Norway

## Abstract

**Background:**

Animal feeding and spawning migrations may be limited by physical barriers and behavioral interactions. Dam constructions (e.g. hydropower) commonly include gateways for fish migrations to sustain ecological connectivity. Relative genetic impacts of fish passage devices versus natural processes (e.g. hybrid inferiority) are, however, rarely studied. We examined genetic (i.e. microsatellite) population connectivity of highly migrating lake-dwelling Arctic char (*Salvelinus alpinus*), introduced 20 generations ago, across and within two subalpine lakes separated by a dam with a subterranean tunnel and spill gates after 7 generations. Due to water flow regime, the time window for fish migration is highly restricted.

**Results:**

Char populations, with similar genetic structuring and diversity observed across and within lakes, were admixed across the dam with fishways during feeding. For spawning, however, statistically significant, but very low population differentiation (θ; 0.002 - 0.013) was found in nine out of ten reproductive site comparisons, reflecting interactions between extensive migration (mean first generation (F_0_) = 10.8%) and initial site fidelity. Simulations indicated that genetic drift among relatively small effective populations (mean *N_e _*= 62) may have caused the observed contemporary differentiation. Novel Bayesian analyses indicated mean contributions of 71% F_0 _population hybrids in spawning populations, of which 76% had maternal or paternal native origin.

**Conclusions:**

Ecological connectivity between lakes separated by a dam has been retained through construction of fishways for feeding migration. Considerable survival and homing to ancestral spawning sites in hybrid progeny was documented. Population differentiation despite preceding admixture is likely caused by contemporary reduced reproductive fitness of population hybrids. The study documents the beginning stages of population divergence among spatial aggregations with recent common ancestry.

## Background

Genetic differentiation, fundamental to population genetics, is initiated by restricted gene flow and reproductive isolation mechanisms within gene pools [1,2]. Habitat and population fragmentation with concomitant disruption of ecological connectivity is threatening biota worldwide, on large scales determined by time since isolation and physical reproductive barriers among demes [3]. On finer scales, genetic differentiation may additionally involve reproductive behaviors and social interactions [4,5]. Addressing genetic effects of physical barriers and reproductive behaviors in novel populations may forecast evolutionary consequences of human intervention.

Natural ecological connectivity may fluctuate in time and space, but anthropogenic alterations (e.g. water regulation) typically exaggerate this variation. Human impacted water flows and water habitat fragmentation expanded greatly worldwide in the twentieth century [6]. In Europe (excluding Russia) close to all large river systems are fragmented by dams [7], inevitably presenting impediments to migration. Construction of habitat corridors to sustain ecological connectivity may in theory counteract negative fragmentation effects [8], although empirical evidence is limited. A number of often retrospective fish passage improvements have been designed to reduce ecosystem wide effects of (hydropower) dams [9]. Dams commonly allow passive or active downstream drift of fish, but obstruct upstream migration. World Commissions of Dams with contributing papers [10,11] and recent reviews [12-14], highlight the need for empirical studies addressing long term effects of fish passage success. Migration barriers may obstruct passage directly or negate otherwise adaptive benefits of homing and assortative mating indirectly [15-17].

Genetic drift and behavior may induce reproductive isolation mechanisms operating on fine scales. Genetic microstructure even within continuous habitat is reported [18,19], although the biological relevance of weak differentiation is debated [20-22]. Within-lake genetic structure for highly mobile fish without obvious migration barriers have similarly been documented [23-28] for a traditional evolutionary time perspective. Genetic structuring and adaptive microevolution may, however, also occur on contemporary time scales [29-33]. Recently founded or invasive species provide opportunities to study initial genetic divergence and evolution in the wild [34], but few studies have evaluated contemporary genetic structure in the invasive species [35-37]. Moreover, we are not aware of assessment of initial genetic structure in recently founded and highly migrating species that are temporally (i.e. seasonally) admixed. Recently founded salmonid species in freshwater lake habitats represent natural small scale experiments that provide opportunities to study the details of early stages of population divergence.

Micro-scale studies under initial admixture introduce challenges concerning sampling design and analytical approaches. For obvious reasons, sample sizes are often restricted both in numbers and temporal replicates. In continuous habitats sampling units may be difficult to delineate. Widely used Bayesian methods to infer cryptic population structure [38-40] may not, however, detect population substructure of recently established populations with low differentiation. Inclusion of population migrants and hybrids (i.e. admixed ancestry) in presumably distinct populations may lead to underestimates of differentiation and erroneous conclusions regarding reproductive isolation.

Reproductive isolation may be reinforced by homing, as documented within a wide range of species [41,42]. Successful reproduction of migrants may be common, caused by relaxed selection against non-natives resulting in non-native offspring, or hybrid offspring with admixed ancestry which break down incipient population differentiation [5,43]. Further differentiation requires reduced reproductive success of non-native and hybrid progeny. Therefore, studies of population admixture and hybrid contribution to population segregation under incomplete divergence are important and may possibly unveil biological relevant differentiation. Traditional measures of population differentiation combined with recently developed Bayesian statistics [44] now permit studies of how inter-population hybrids may affect population differentiation.

**Figure 1:**
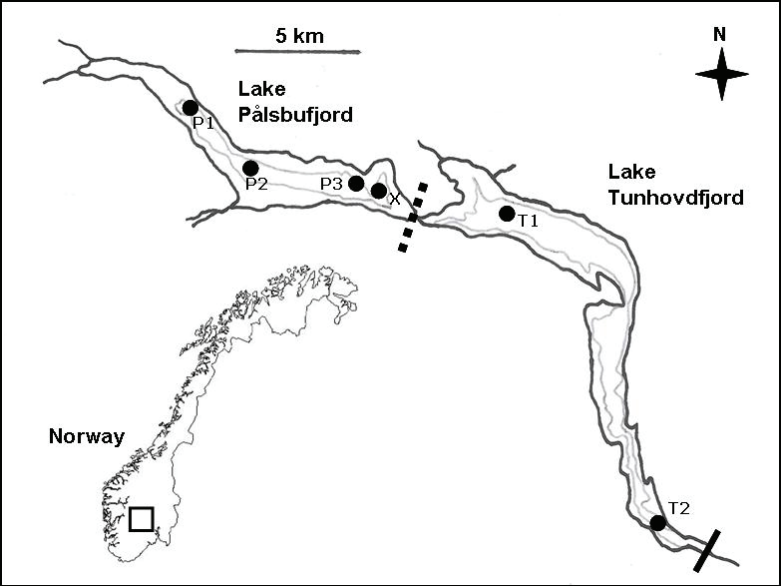
**Map of sample localities**. Map of the study area with Lake Pålsbufjord (PAL) and the downstream Lake Tunhovdfjord (TUN). Minimum water level (LRV) is indicated by the grey line, and sampling sites are marked with dots and abbreviations (P1 - 3, T1 - 2 and X). Solid bars indicate semi-barrier, while broken bars indicate partial restriction to up- and downstream migration.

Here we study genetic differentiation of Arctic char (*Salvelinus alpinus*), introduced around 1920 (i.e. 20 generations ago), across and within two lakes: Pålsbufjorden (PAL) and the downstream Tunhovdfjorden (TUN) (Figure 1). The two lakes, naturally connected by a short river, were separated by a dam in 1946, creating two hydropower reservoirs. Both lakes have geographically distinct char spawning sites (PAL; P1 - 3, TUN; T1 - 2) for which we assess population connectivity implementing 10 microsatellites. The first objective was to examine genetic structure across lakes to quantify present effects of two migration enhancements at the dam; a subterranean tunnel and spill gates. An additional summer sample (X) of lake feeding and presumably admixed char from PAL was included to assist interpretation of differentiation. We expected to find greater genetic differentiation across lakes than across populations within lakes, and assignment of sample × to PAL populations. We also expected that passive migration downstream would exceed upward migration and result in observed asymmetric migration and associated genetic variation. The second was to examine migration patterns and population hybrid contributions potentially responsible for contemporary genetic substructure. Due to expectations of low population differentiation in the progress towards reproductive isolation, we intend to deliberate over the paradox of population divergence in the face of migration, rather than to state absolute assertions.

## Results

Amplification and allele calling of 190 individuals over 6 sites was obtained in 99.8% of the cases, and secondary amplifying and allele calling of a subsample gave consistent results. Number of alleles per locus averaged 15.4 (SD ± 7.2, range 2 - 26, Additional file 1). Quality control screening did not reveal indications of scoring error due to stuttering or evidence for large allele dropout. The test for null alleles assuming Hardy-Weinberg equilibrium (HW) suggested that null alleles may be present for locus *Sco204 *in sample site T2, by general excess of homozygotes. However, the combined probability for all homozygote class frequencies was not significant (P > 0.05). No deviations from HW were found, but nonrandom association of alleles from different loci was indicated in 12 and 9 (*Sco 213/Sfo8, Smm17, Smm24, Str73; Sfo8/Smm17, Smm24, Str73; Smm24/Smm17, Str73*) of 45 tests uncorrected and after false discovery rate (FDR) correction [45] at 5% level, respectively. Genetic variation revealed a mean value of local F_ST _= 0.024 (range 0.013 - 0.036), and inbreeding F = 0.028 (range 0.003 - 0.053) over sites.

### Dam barrier to migration and gene flow

Measured lake water levels and estimated velocities in the fishways indicated possible upstream migration only within very limited windows of time. During 2000 - 2010, migration through the tunnel was feasible in less than 3.6% of the year (mean 13 days per year, SD ± 20, min 0, max 55). Migration through the spill gates was possible in less than 1.7% of the year (mean 6 days per year, SD ± 4, min 1, max 16). Upstream migration was possible in all seasons through gates, but only through the tunnel in spring.

Observed heterozygosity and allelic richness was similar between lakes (Additional file 1). Number of private alleles found totaled 22, 6 in TUN and 16 in PAL (data not shown).

The total allele frequency variation consisted of 99.3% (P < 0.001) variation within populations, 0.7% (P = 0.001) variation among spawning sites within lakes and no variation between lakes (P = 0.499). Genetic differentiation of char from the two connected lakes PAL and TUN was, however, highly significant (P = 0.001) but very low (θ = 0.003, standardized (θ') = 0.014). Removal of first generation migrants (F_0_), amounting to 7 and 9% in PAL and TUN (not significant after correction, 170 tests, threshold P = 0.0003), respectively, did not affect this differentiation. STRUCTURE did not detect genetic structure among lakes or sites (K = 1).

Estimated F_ST _and exact tests revealed that one spawning population (T2) was significantly different from a summer feeding sample (X) in PAL (Table 1). Sample × assigned to both lakes (TUN, 25%), and 30% was most similar to spawning site T1 on a population level (Table 2), indicating upstream migration.

**Table 1:** Population differentiation

Sample site	P1	P2	P3	X	T1	T2
P1		0.0125*	0.0051*	-0.0046^NS^	0.0076*	0.0060*
P2	0.0035*		0.0038*	0.0044^NS^	0.0022*	0.0105*
P3	0.0103*	0.0050*		-0.0045^NS^	0.0030*	0.0098*
X	0.6572^NS^	0.1290^NS^	0.9587^NS^		< 0.0001^NS^	0.0026*
T1	0.0085*	0.0473^NS^	0.0019*	0.5535^NS^		0.0046^NS^
T2	0.0004*	< 0.0001*	< 0.0001*	0.0045*	0.0580^NS^	

Pairwise test of differentiation (θ, upper), and P-values from exact test in TFPGA (lower) between spawning char in PAL (P1 - 3), TUN (T1 - 2) and a mixed summer sample in PAL (X). Indicative adjusted nominal level (5%) in pairwise test for multiple comparisons is 0.00333, initial k = 15. Non-significant (^NS^) and significant (*) differentiation at 5% level after FDR correction.

**Table 2:** F_0 _migrants and population assignment

					Assigned population			
			
Sample site	N	F_0_	P1	P2	P3	T1	T2	na
P1	34	0.15	0.32	0.15 [0.03]	0.32 [0.03]	0.18 [0.06]	0.00	0.03 [0.03]
P2	34	0.03	0.12	0.38	0.38 [0.03]	0.12	0.00	0.00
P3	34	0.12	0.03	0.09 [0.03]	0.62	0.15	0.03	0.09 [0.09]
T1	34	0.09	0.18 [0.03]	0.12 [0.03]	0.21 [0.03]	0.38	0.09	0.03
T2	34	0.15	0.21	0.09	0.24 [0.09]	0.35 [0.06]	0.12	0.00
X	20		0.15	0.00	0.55	0.3	0.00	0.00

Frequencies of first generation (F_0_, before FDR correction) migrants and char from the spawning sites (PAL; P1 - P3 and TUN; T1 - 2) and a mixed summer sample in PAL (X) assigned (na: non-assigned) to the five spawning sites estimated in GENECLASS. Frequencies of F_0 _migrants are given in brackets.

Migration beyond F_0 _was assessed with a maximum likelihood approach for assignment and with Bayesian estimates for present and contemporary estimates. Self-assignment of individual char in GENECLASS2 revealed 93 and 46% correct assignments to Lake PAL and TUN, respectively, suggesting a downstream source-sink structure. Probability estimates of ancestral origin over the last generation estimated in the software program BIMR, also indicated high, but more symmetric migration rates. With the two lakes as populations, mode allele frequencies showed almost equal ancestral native origin for the two lakes (PAL; 56%, highest posterior density intervals (HPDI): 34 - 75, TUN; 59%, HPDI: 25 - 90), with estimated HPDI for non-native origin of 24 - 65% and 9 - 74%, in PAL and TUN respectively.

Mode probability of hybrid ancestral origin for the pooled samples within lakes estimated in BIMR were 50% in PAL (HPDI = 36 - 49) and also 50% (HPDI = 13 - 48) in TUN. Nevertheless, pure origin from the home lake was more common than pure origin from the other lake.

### Lentic population migration and admixture

All except one test of genetic differentiation among spawning sites were significant (mean θ = 0.007, SD ± 0.003). Mean standardized differentiation (θ') was 0.030 (SD ± 0.016). Exact tests corroborated the pattern of significantly differentiated spawning sites (Table 1). Using spawning sites as prior populations, 37% of the char assigned to its sampled site, varying from 12 - 62% among sites (Table 2). Mean probability of best assignment, however, was only 51% (SD ± 28), indicating recent admixture, migration, or un-sampled populations. Exclusion of 18 char (11%) found to be F_0 _migrants across spawning sites (range 1 - 5, Table 2, not significant after correction, 170 tests, threshold P = 0.0003) increased population segregation and resulted in all spawning populations being significantly different (θ > 0.002, θ' > 0.008, P < 0.021). This finding corroborates the estimated migration rates. Analyses in IMMANC verified migrant detection (8%) with a power of 0.968. The Bayesian probability that the sampled alleles within spawning sites originated from the same site last generation (ancestral rate) were 0.47 across sites (SD ± 0.09, min 0.34, max 0.60), and higher than the probability of origin from any other population (mean 0.13, SD ± 0.10, min 0.02 max 0.29).

**Figure 2:**
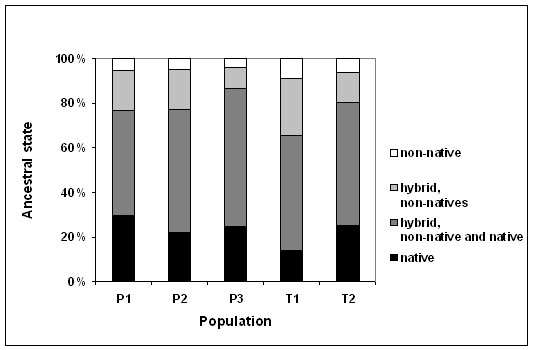
**Ancestral origins**. Pooled ancestral state proportions of spawning char at site P1 - 3 in Lake Pålsbufjord, and site T1 - 2 in Lake Tunhovdfjord, estimated in BIMR. Hybrid native indicates that one parent is from the local population, while hybrid non-native indicate parental origin of two different non-native populations.

Mixed ancestry analyses indicated that population hybrids constituted a substantial proportion within spawning sites. The majority of hybrids within all sites were progeny of native and non-native char (mean 54%), whereas hybrids of two non-natives were less common (mean 17%, Figure 2). Similar to pooled lake estimates, the mode probability of home origin within sites was consistently higher than pure non-native origin within sites, and consequently higher for hybrids with partial native ancestry than for any other mixed ancestry across sites. Standard deviations of mixed ancestry estimates were generally similar to mode estimates.

### Potential interpretation bias

Potential bias and biological relevance of observed data were addressed to assure sound interpretations. F_ST _tests excluding loci with possible LD (*Sco213 *and *Sfo8*) revealed similar differentiations as for all loci combined (data not shown). The F_ST _estimate per locus averaged 0.0064 (SD ± 0.006, min 0.001, max 0.023). Exclusion of single loci generally weakened spawning site differentiation, but did not change the above pattern, thus indicating neutral behavior of markers.

AMOVA analyses segregated based on year-classes revealed 100% variation within sites and a lack of variation among year-classes (F_ST _= -0.001, P = 0.624), whereas hierarchical analyses based on spawning sites revealed that 99.45% of genetic variation stemmed from within-population variation and 0.55% (F_ST _= 0.006, P = 0.002) from among-population variation. Thus, genetic variation was congruent with the high variability of microsatellites used, indicating temporal stability among consecutive year-classes. Pairwise year-classes were not significantly different within any spawning site (θ < 0.060, P > 0.013), or pooled across sites (θ < 0.004, P > 0.184). Removal of year-classes one-by-one changed the significance in 15.5% of the pairwise tests, but did not affect the pattern of differentiation among spawning sites.

**Figure 3:**
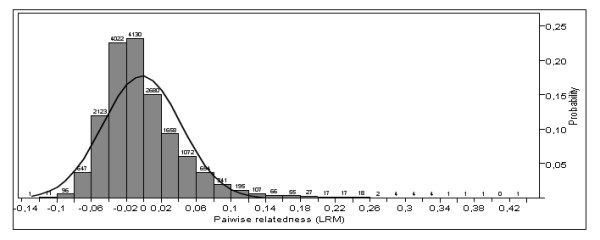
**Pairwise relatedness**. Ritland and Lynch (1999) pairwise relatedness (LRM;[153]) histogram for the pooled sample, with fitted normal density curve. Pairwise counts are given on top of bars.

We did not find evidence for family structures affecting population differentiations. Simulations indicated a power of 1.000 and 0.918 to discriminate unrelated from full sib and half sib, respectively. Only 0.1% of pairwise tests had relatedness coefficients ≥ 0.25, indicating half sib relationship, and half of these were across sites. Mean distribution of relatedness (LRM) for the pooled dataset was negative (μ = -0.003, SD ± 0.045, Figure 3), although positively skewed (1.640) and not normally distributed (P = 0.010). Sites individually revealed the same pattern (not shown), with lack of bimodal patterns that could have indicated kin-groups.

Effective population sizes (*N_e_*) were estimated to be 52 - 71 individuals within spawning sites, 133 - 142 within lakes, and 233 in total (Table 3), supporting both across and within lake genetic structure. Estimates were not affected by prior values of *N_e_*. Estimates of effective parental population size (*N_b_*) were generally higher but similar to *N_e_*, except negative and infinite for population P1. This may indicate sampling error, or no evidence for LD caused by genetic drift due to a finite number of parents. Tests for LD did not, however, give significant deviations for any loci combination at this site, after FDR correction. Recent bottlenecks over all loci were not confirmed for any of the tests (P > 0.080), assuming a mixed mutation model (TPM). Neither did tests for di-nucleotide and tetra-nucleotide loci separately, under TPM and a single-step mutation model (SMM) respectively, reveal signs of severe loss of allelic diversity (normal L-shaped distribution, P > 0.060), except for population P2 and tetra-nucleotide loci, where the Wilcoxon test for heterozygosity excess was significant (P = 0.030). Test for isolation by distance (IBD) was positive but not significant (r = 0.29, P_upper _= 0.250, P_lower _= 0.760).

**Table 3:** Effective population sizes

Lake	Population	*N_e _*(ONESAMP)	*N_b _*(LDNE)
PAL	P1	63.6 (47.9 - 106.8)	∞ (229.3 - ∞)
	P2	53.9 (41.3 - 87.5)	58.0 (43.3. - 84.0)
	P3	51.7 (39.9 - 83.0)	56.5 (42.8 - 79.8)
	PAL total	132.6 (111.0 - 173.5)	272.5 (198.7 - 416.5)
TUN	T1	69.8 (51.8 - 124.0)	62.0 (46.2 - 90.3)
	T2	70.6 (48.8 - 135.1)	45.4 (33.3 - 66.8)
	TUN total	142.4 (100.3 - 263.6)	105.6 (79.9 - 149.5)
PAL/TUN	total	232.5 (202.7 - 278.9)	312.6 (244.6 - 422.0)

Estimated mean effective population size (*N_e_*) and parental population size (*N_b_*) with 95% C.I., from ONESAMP and LDNE respectively from PAL (P1 - 3) and TUN (T1 - 2) and pooled data. Negative estimates are reported as infinite (∞).

**Figure 4:**
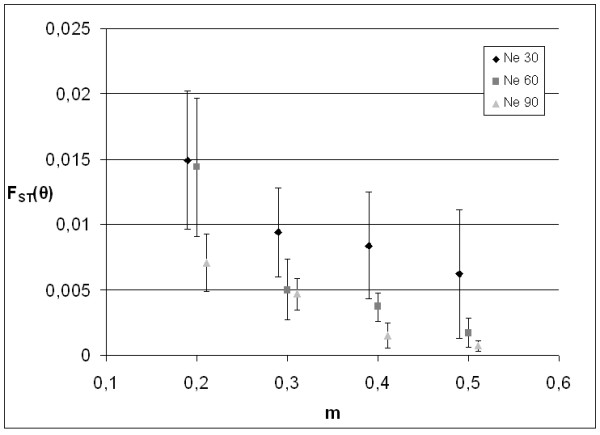
**Population differentiation simulation**. Simulated effects of relevant migration rates and effective population sizes to mean (SD) pairwise differentiation between 5 populations, after 20 generations.

Forward simulated F_ST _estimates (Figure 4), indicated that genetic drift alone may have caused the observed population differentiation. For instance, five populations with *N_e _*= 60 were significantly differentiated (mean θ = 0.005, SD ± 0.002, P < 0.047) after a mean migration rate of 0.3 in 20 generations, assuming initial admixture after introduction. Ten independent replicates of this scenario indicated similar differentiation (mean θ = 0.006, SD ± 0.001). IMMANC indicated detection power for migrants (8%) and hybrids (7%) in our sampled dataset to be 0.968 and 0.638, respectively.

A forward simulated alternative hypotheses of m = 0.01 revealed high differentiation (mean θ = 0.122, SD ± 0.019, P < 0.001) between reproductive sites, whereas m = 0.99 revealed lack of differentiation (mean θ = 0.001, SD ± 0.001, P > 0.176). A scenario without migration only indicated 5.0% (1.3% after correction) F_0 _migrants in GENECLASS, similar to expectations from type 1 errors. Similar analyses in IMMANC indicated 10.7% (1.0% after correction) F_0 _migrants and 10.0% (1.3% after correction) F_1 _hybrids, with mean power > 0.999. Coalescent simulations supported that drift alone may have caused contemporary site fidelity (Additional file 2).

## Discussion

### Migrations

Our results suggest that constructed gateways for migration ensured alternate habitat utilization and gene flow across the dam separating the two studied lakes. Significant differentiation was found between spawning char from the two interconnected lakes, but differentiation among populations within lakes equaled or exceeded that for lakes. Without assuming any direct relationship between F_ST _and gene flow (below), these relative measures are low and indicate high connectivity across the dam. Char with genetic assignment to distinct populations in both lakes were caught during feeding migration in the upper lake in summer, indicating upstream migration across the dam. Corroborating this, gene diversity and genetic structure were similar across and within lakes.

Extensive migration reflecting seasonal foraging admixture across lakes and populations is independently corroborated by tagging studies [46,47]. Barrier type, hydrology and life stages of focal species all interact in determining the impact of barriers [48]. Tunnels associated with turbine outflow at dams are known to attract ascending salmonids [49-51]. The lack of turbines in the PAL dam tunnel makes migration through the tunnel possible. It is an open question, however, to what extent char in our study lakes utilize the spill gates or the tunnel for (upstream) migration. Ongoing studies in brown trout also reveal high dispersal across the focal dam, with unknown proportional use of the fishways (unpublished). Regardless, results indicate that char, in both cases, take advantage of the highly restricted time window suitable for upstream migration. Disentangling present from previous genetic structure and biological interpretation of low differentiation is, however, challenging (below), and results should therefore be interpreted with caution.

### Contemporary genetic substructure

The present study documents that genetically differentiated sympatric populations can be established within decades. Pairwise genetic differentiation was found to be significant, but very low, both among lakes and among most spawning sites within lakes, indicating spatial reproductive isolation even within continuous habitat. Estimates of *N_e _*supported this. However, STRUCTURE did not reveal any population structuring. Performance of Bayesian clustering methods decreased for F_ST _< 0.02 [52], and we propose that even the novel algorithm used in STRUCTURE [39] may not detect recently established populations with very low differentiation (F_ST _< 0.01).

Low differentiation is expected due to the short time since species introduction, and the migratory behavior of char. Following the simplistic [53,54] equation F_ST _≈ 1/4*Nm *+ 1), our mean local F_ST _estimates indicate long term *Nm *= 10.2, and migration rates of 0.17 assuming *N_e _*= 60. Fixed *m *= 0.5 would reveal the same differentiation if *N_e _*is as low as 20. As an example, 60 individuals and *m *= 0.5 result in the same differentiation as e.g. 1000 individuals with 3.0% gene flow. In contrast, if we assume F_0 _analyses (mean *m *= 0.11) are representative for present gene flow, and implement *N_e_*'s of 60, the present *Nm *is 6.6, likely overestimated due to common reproductive inferiority in migrants. Consequently, mean gene flow between populations may have decreased since introduction. Thus, present genetic structure does not reflect panmixia [55]. Both estimates, however, indicate that gene flow is too small to bring about drift connectivity [53], but too large for Bayesian detection of subdivision [54]. A denotation of the sampled reproductive entities as populations, according to the evolutionary and especially the ecological paradigm [22] is, however, debatable. Maximum F_ST _estimates will be limited by heterozygosity [21], and estimated differentiation may underestimate true population differentiation [56], especially when gene diversity is high [57]. Similar standardized population differentiation is previously found in char [58]. Direct comparisons of observed θ are complicated, but similarly low and biological relevant differentiations are found in both mammals and fish [59,60]. Interestingly, similarly low, but significant differentiations are also found among populations of char founded thousands of years ago [61], and population structure of lake-dwelling brook char (*Salvelinus fontinalis*) has been found on a smaller geographical scale under migration-drift equilibrium [62] than in our study. Low but significant pairwise differentiations are also found in invading catadromous crustaceans separated after recent colonization [35]. This study also documented strong genetic drift simultaneously with significant differentiation (F_ST _> 0.007), but among year classes, as opposed to our study.

True substructures in our study were corroborated by *N_e _*estimates, genetic variability, year-class stability and low number of sib-groups within sites. Invasive species, such as char in our lakes, are expected to express low genetic variation and *N_e_*, caused by founder events and population bottlenecks [63-65]. The high level of genetic variation within the examined loci in our populations does not suggest depauperate populations or bottlenecks on the surveyed scale, which would otherwise certainly support genetic differentiation caused by strong genetic drift in very small founding populations. The concept of founder populations, however, suggests that current structure is novel. We are not aware of evidence from the literature that fish populations bred in admixture may resume historic population structures in novel habitat. Simulations, however, documented that drift alone, even in larger populations, could cause rapid differentiation. Estimation of population size based on single samples relies on a number of simplifying assumptions [66,67]. Our estimated population sizes should therefore be interpreted with caution. Echosounder and gillnetting experiments [68] suggest a population size of char around 9000 in PAL, returning *N_e_*/*N *≈ 0.015, which is common for marine fish [60] but low compared to most salmonids [69,70]. Nevertheless, estimated *N_e _*and *N_b _*gave similar results, and suggest population sizes sufficient to confer contemporary genetic stability as well, as opposed to a transitory or strict metapopulation structure [71]. It is possible, however, that estimates are inflated by immigrants and sampling of hybrid zones, even though no deviations from HW were evident. Regardless, the question remains whether one is actually able to sample biological populations correctly, and if hybrid contribution represents true population structure. The presence of immigrants and hybrids, not contributing to the reproductive population gene pool, can easily complicate interpretations of genetic diversity, *N_e _*and genetic differentiation.

### Initial genetic structure

The low level of interspecies competition and vacant niches fits well with rapid establishment of char in our lakes, and zooplanktivores such as char show a high rate of invasion success [72]. Genetic differentiation by means of the evolution of reproductive isolation can be viewed as non-ecological modes of differentiation such as founder effect or genetic drift in small populations, or by divergent natural selection [73,74]. Lag time between initial colonization and the onset of rapid population growth is expected in invasive species [75], in particular if evolutionary adaptation is important during colonization. Thus, the rapid expansion in the lakes surveyed [76] corroborates the independent simulations, pointing to genetic drift as an initial diversifying factor for observed population differentiation. However, theory [1,77,78], experiments [79] and empirical studies [80-85] all suggest a potential for rapid evolutionary changes involving adaptation in newly founded populations. Moderate levels of plasticity are also typical for char [86,87], and may also have been optimal for fast genetic evolution [81,88-90].

The combined genetic and ecological data suggest that the initial genetic structure and diversity within our lakes is a result of site fidelity and initial drift. Several evolutionary forces may, however, interact at the same time. Realistic and rapid drivers of cryptic kin selection [91] or discontinuous adaptive variation [32,92-94] may add to incipient reproductive isolation. Once sub-structure is established, site fidelity, drift, and low effective migration rates may strengthen differentiation in time and space. Such isolation by adaptation [95] is likely facilitating drift in neutral loci by reduced gene flow as a general barrier mechanism [96], and even neutral markers may detect adaptation in the face of intermediate migration [97].

### Differentiation despite extensive migration

Substantial migration among reproductive sites was found, without significant IBD, suggesting little present drift. Thus, although common in salmonids, equilibrium between gene flow and genetic drift is not present [98,99]. Migration analyses may be biased by method assumptions, un-sampled populations, low differentiation and convergence problems [44,100,101]. However, all methods used to interpret migration in our study, indicated considerable migration among lakes and populations. Extensive migration among populations is often seen in salmonids [43]. Homing and kin discrimination will, however, contribute to genetic structuring and is widely known in fish [102], particularly char [103-106]. Pairwise genetic differentiation of other neighbouring lacustrine char populations has been found to be highly significant, despite long term migration rates of 1.853 - 2.755 individuals per generation [107]. Low levels of effective migration, i.e. gene flow among sympatric populations, contrary indicate breeding site fidelity in established populations [107,108]. Reproductive divergence despite extensive migration in the study at hand indicates restricted effective gene flow between lakes and populations, even after 20 generations. Thus, at first glance, one may be tempted to conclude that results indicate reproductive selection against immigrants in newly established populations, along the lines of previous studies [77,83,109]. Alternatively, one may speculate that breakdown of non-native gene flow is caused by reduced reproductive fitness of population hybrids.

### Hybrid contribution during naturalization

The hierarchical Bayesian analyses of mixed ancestry confirmed non-native spawning success. The large proportion of hybrids documents reproduction by immigrants, i.e. relaxed selection against non-natives early in differentiation and survival of hybrids. Selection against population hybrids and immigrants strengthen divergence [110,111]. Hybrids of sympatric and closely related (F_ST _= 0.070) perch (*Perca fluviatilis*) were found to have reduced fitness in early life stages in laboratory experiments, but the authors did not test for hybrid inferiority in vivo [112]. Novel habitats without competition as in our study may have promoted survival of hybrids, despite potentially reduced fitness. However, hybrids may be less competitive in secondary stages of naturalization, as increased population divergence reduces hybrid fitness [113].

In our study, hybrids without parental origin from their spawning site (Figure 2; non-native hybrids) constituted a minor part of mature char caught at their reproductive site. The considerably larger proportion of individuals with partial native ancestral origin indicates hybrid homing. Reproductive units sustained by kin discrimination are found in a range of species [114-117], and hybrid juveniles of char will be in close proximity to kin when hatching. Population differentiation, however, could only be established if reproductive success of returning hybrids is low. Admixed individuals would otherwise have caused migration loads obscuring effects of selection and differentiation [109]. Postmating reproductive isolation and hybrid inferiority have been addressed for a century [2,111]. Both theoretical and empirical studies frequently reveal selection against hybrids during speciation, although its origin seems unclear [74,96]. Sexual selection (mate choice) against hybrids is found across taxa [e.g. insects; [118], fish; [119]], and may also characterize char. Such mechanisms may evolve faster in small populations [120], as within our sites. Social recognition of relatives in fish using odour cues can induce assortative behavior in fish [121-123]. Finally, high dispersal rates may increase the absolute number of hybrids, but the increased competition reduces hybrid reproductive fitness, and consequently increases reproductive isolation of the resident population [96].

## Conclusions

Migration corridors between populations separated by dams may be valuable for sustaining evolutionary potential. This study demonstrates that even temporally very limited connectivity through a subterranean tunnel and spill gates between two regulated lakes, likely counteract genetic isolation in char.

Initial stages of genetic divergence of subpopulations, despite high migration rates, are documented in this study. We are not aware of previous studies addressing hybrid migration and reproductive success in recently founded and admixed populations. Our study indicates that population differentiation may be detectable, despite inclusion of hybrid sub-populations, when assessing genetic structure among populations. Combined also with independent mark-recapture data by Aass [46,47], our genetic approach indicates that hybrids contribute extensively to migration rates in recently founded populations. Population hybrid events may have been important in establishing the diverse population structure in the novel habitat [78]. However, present differentiation implicates nascent non-native and hybrid inferiority, whether biologic or ethologic [32,91]. Estimates generally gave wide HPDI, an inherent problem expected from low differentiation affecting accuracy [44], and results must be interpreted with caution.

Few population genetic software programs have been evaluated with respect to their performance in detecting low genetic differentiation, making interpretation of differentiation close to admixture difficult [53,124]. However, restricted power in admixture analyses should not restrict studies of hybridization mechanisms, as they raise consequential questions in micro-evolution and behavioral ecology. Both small scale empirical and larger simulation studies of incomplete reproductive isolation may guide management of invasive and naturalized species, potentially unveiling initial population differentiation mechanisms.

## Methods

### The study species

The Arctic char is a salmonid fish with Holarctic distribution [125] showing extreme phenotypic and life history variation, exemplified by weight at maturation from 3 g to 12 kg [87]. This reflects the species capability to evolve trophic polymorphism and possible genetic differentiations within drainages [126] and lakes [107,127,128]. While most populations of char result from natural postglacial colonization, high altitude populations are introduced by man. When in sympatry, Arctic char typically have a benthopelagic distribution in landlocked habitat. Young char typically feed in the littoral, shifting towards a highly migratory pelagic feeding pattern as adults. Lacustrine char are commonly 20 - 40 cm in length and a few years old when mature, and spawn in the littoral within a few weeks in autumn. Site fidelity is common, but reproductive ecology is poorly understood (see Jonsson *et al*. 2001 [86], Klemetsen *et al*. 2003 [87] and Johnson 1980 [129] for excellent reviews).

### The study site

Lake Tunhovdfjord (TUN) and Lake Pålsbufjord (PAL) are part of a 35 km long sub alpine hydroelectric reservoir located in south-central Norway (48°E, 67°N, Figure 1), regulated first in 1919. A hydropower dam separating the two lakes was erected in 1946, restricting the previously free migration among the lakes to migration through spill gates in the dam (lower level 734.9 m a.s.l., c 10 m, c 3.1 m^2^, neutral gradient) and a subterranean anthropogenic branch (lower level 722.4 m a.s.l., 1300 m, 7.1 m^2^, neutral gradient) with outflow 600 m below the dam. Lake PAL now has a surface area of 5.25 - 19.5 km^2 ^with maximum depth 25 m, 725.5 - 749.1 m a.s.l.. Lake Tunhovdfjord, located immediately downstream, has a surface area of 14 - 25 km^2 ^and a maximum depth 70 m, 716.4 - 734.4 m a.s.l. Arctic char are sympatric with brown trout (*Salmo trutta*) and invasive European minnow (*Phoxinus phoxinus*) in both lakes. Brown trout have probably been native for > 6000 years [130,131]. Minnow were introduced coincidentally around 1920.

In 1910, 10.000 fry from a natural char population in Lake Tinnsjø (187.2 - 191.2 m.a.s.l., 51 km^2^, mean depth 190 m), situated in a different watershed 80 km south-west of PAL and TUN were stocked in two alpine lakes above PAL [132,133]. Char were first observed in PAL in 1919. The population expanded rapidly and was present in large numbers in both PAL and TUN after a decade [76]. Densities have thereafter been highly variable, probably because of large water level fluctuations and associated egg mortality [68,134]. Char in these lakes seldom become more than 8 years old and have a generation time of about 4 years (20 generations since introduction). They are usually < 200 g [68], although cannibalistic individuals of 3 kg have been reported [47]. Extensive mark-recapture studies have revealed substantial char movements within and between the lakes [134], even after the construction of the dam in 1946. Char feeding migration intensity increases in late summer and autumn. The majority of adult char congregate in outlets and narrow parts of the lakes after spawning in autumn when water level drops, followed by c. 10 - 50% passive displacement downstream in the spring [134]. Char emigrating PAL often attempt to re-enter the lake, but successful upstream migration is unknown [47].

Arctic char are low performance swimmers as opposed to brown trout [135]. Maximum relative swimming speed (body length s^-1^) is about 2.8 [136], thus a water velocity of 150 cm s^-1 ^is likely an absolute limit for upstream migration for studied char across the dam. The Mannings formula; v = M * R_h_^2/3 ^* √I [137], assuming M = 35, was used to estimate elevation heads associated with 150 cm s^-1 ^water velocity in the fishways. The water level below the dam in TUN commonly increases (up-arches) to the lower level of the gates. There is also free entrance from TUN to the subterranean tunnel independent of water level. Thus, migration was estimated to be solely restricted by water levels in PAL at 734.9 - 735.2 m a.s.l. in the gates, and 722.4 - 726.4 m a.s.l. in the tunnel. The water level in PAL was registered from year 2000 - 2010.

### Sampling

A total of 190 mature char (mean total length 244 mm, SD ± 31, range 170 - 334) were sampled by gillnetting at four sites in PAL and two sites in TUN (Figure 1). Samples represented 54% males (i.e., presence of gametes) and the year classes 1999 - 2003 (N = 31, 37, 48, 37 and 2, respectively, as determined by scales and otoliths of 155 char). All char except those from T2 were aged, but these fish had similar size distribution. Samples from site P1 - 3 and T1 - 2 each included 34 char ready to spawn this season (gonadal stage 4 - 6) caught at traditional and geographically separated spawning sites below minimum water level (LRV) during spawning time, and are therefore treated as discrete populations, although true population boundaries are unknown. Additional spawning sites within the two lakes are not known. Site × (N = 20) is a randomly chosen mid-lake sample from late summer two months prior to spawning, representing admixtured non-spawning char.

### Microsatellite genotyping and variability

Tissue samples of approximately 2 mm2 from the adipose fin were preserved in 96% ethanol in the field, and DNA was isolated using DNeasy kit (QIAGEN), following the manufacturer's guidelines. Microsatellite polymorphism was analyzed by means of 10 di- and tetra-nucleotide loci known to be polymorphic in S. alpinus; Mst-85 [138], Sco202, Sco204, Sco213 [139], Sfo-8, Sfo-23 [140], Smm-17, Smm-24 [141], Ssa-85 [142] and Str73 [143]. One primer for each locus was end-labeled with fluorescence (HEX, FAM and NED), and run partly as multiplex PCR reactions; multiplex A; Sfo-8, Smm-17, Smm-24, Str73 and Sco21, multiplex B; Sco202 and Sco204, and multiplex C; Sfo-23 and Ssa-85. Each PCR contained 2 μl genomic template DNA and 8 μl reaction mixtures containing 1 - 2 pmol primer, 50 mM KCl, 1.5 mM MgCl, 10 mM Tris-HCl, 0.2 mM dNTP and 0.25 U Taq polymerase enzymes (Ampliqon). Thermocycling parameters after denaturation at 95°C for 2 min were 24 - 34 cycles of 95°C for 30 sec, annealing temperature of 55°C for 30 sec, followed by an extension at 72°C for 45 sec. The last polymerization step was extended to 10 min. PCR products were added to buffer containing formamide and labeled standard (ROX Std 400, Applied Biosystems), and electrophoresed using an ABI Prism 3100 Genetic Analyzer (Applied Biosystems). The software GENEMAPPER v.3.7 (http://www.applied-biosystems.com) was used to score alleles, and all automated allele calling were controlled for by manual reading. Two positive controls were done in each run, and scoring was repeated twice for several loci, and for all private alleles, to check for consistency.

Quality control screenings were performed by testing for null alleles, large allele drop-outs and scoring errors in MICRO-CHECKER v.2.2.3 [144]. The program TFPGA v.1.3 [145] was used for descriptive statistics (number of alleles, observed and expected heterozygosity). Allelic richness was compared among lakes and spawning sites based on minimum sample size for each comparison separately in FSTAT v.2.9.3.2 [146]. Departure from Hardy-Weinberg (HW) via separate one-tailed tests for heterozygote excess and deficiency for each locus in each site was tested by 60.000 randomizations, and a linkage disequilibrium (LD) test between pairs of loci across populations was performed by 450.000 permutations, both in FSTAT. Local F_ST _(diversity standardized) and inbreeding coefficient F were calculated in BIMR v.1.1 [44], including 95% C.I., the F-model [147] and default values, except burn-in and sample size of 10^5 ^iterations, to ensure convergence.

### Genetic structure and temporal stability

Possible genetic segregation among lakes and sites and their significance were tested with the F_ST _analogue θ [148] and pairwise test of differentiation in FSTAT, and by exact test (Raymond and Rousset 1995) in TFPGA, all with 15.000 permutations. The estimated θ was chosen since it out-performs other F_ST _analogues in detecting recently established reproductive isolation [149], and marker neutrality was addressed by jackknifing F_ST _estimates over loci and populations. Standardized measures of genetic differentiation (θ' [150]) were calculated using the software RECODEDATA v.0.1 (http://www.bentleydrummer.nl/software), to address F_ST _estimates dependence on the level of genetic variation. Finally, STRUCTURE v.2.3.2 [39] was used to test whether population differentiation was detected in a Bayesian model based algorithm. The admixture locprior model with correlated allele frequencies was run for K = 1 - 10 with a burn in of 200.000 MCMC steps, followed by 500.000 steps in 10 replicates.

Temporal substructure stability was tested for by possible year-class variation, sib-ship analyses, effective size estimates and for isolation by distance (IBD). Allele frequency variation was tested in a hierarchical fashion (AMOVA; among versus within populations), and compared with an AMOVA with segregated year-classes (among versus within year-classes), to test for temporal stability within spawning sites where age was available (P1 - 3 and T1). Analyses were performed in ARLEQUIN v.3.1 [151], under the infinite alleles model (IAM), standard model, unknown gametic phase and 10^4 ^permutations. Year-class variation was further controlled for by testing pairwise differentiation among year-classes, and excluding one-by-one year-class from site wise θ tests (above). KININFOR v.1.0 [152] simulated the power of applied markers and observed allele frequencies to discriminate between unrelated and full-/half-sib individuals. We ran the simulation with a prior Dirichlet distribution of 1 for Δ_0_, Δ_1 _and Δ_2_, with a 0.05 confidence level, a precision level of 0.01 and 10^6 ^simulated pairs of genotypes. Family structures within sites, possibly affecting population differentiation estimates, were evaluated in GENALEX v.6.2 [153] by the algorithm of Lynch and Ritland [LRM; 154]. Normality of pairwise relatedness was evaluated in JMP v.8.0 [155].

Contemporary methods were used for estimating effective population size (*N_e_*) and effective parental population size (*N_b_*) within lakes and spawning sites from genetic data. Estimates of *N_e _*using summary statistics and approximate Bayesian statistics were computed in ONESAMP [66], after 50.000 iterations, using prior *N_e _*of 4 - 600 for individual sites and 10 - 2000 for the lake samples. Computations were repeated three times, reporting the median result among tests. Runs with prior values ranging from 2 - 1000 were compared to check for consistency and convergence. Parental *N_b _*was similarly estimated based on linkage disequilibrium using LDNE v.1.31 [67]. The model is assuming closed populations, and is eliminating the possible bias on small sample sizes. We used 0.02 as the lowest allele frequency used in the computation to balance bias and precision [156], including a parametric 95% C.I. in the random mating model. The program BOTTLENECK, v.1.2.02 [157] was used to test for recent bottlenecks using the Wilcoxon test and mode-shift tests with 10^4 ^replications, with the two phase mutation model (TPM, including 10% infinite alleles model) and the single step mutation model (SMM).

Presence of IBD, indicating equilibrium conditions, was addressed with a Mantel test of correlation between pairwise θ and linear geographic distance among sites in TFPGA, after 1000 iterations.

### Migration and admixture

A combination of individual based maximum likelihood methods and population based Bayesian methods were employed to overcome the potential challenge of detecting gene flow under low differentiation. First generation migrants (F_0_) were in GENECLASS v.2.0 [158] identified to evaluate migrants influence on genetic differentiation, using the test statistics L_home/L_max and L_home between lakes and spawning sites, respectively, to account for un-sampled populations and maximizing analyses power. Migrants between spawning sites were assigned to the population with highest (> 5%) self-assignment probability. Estimates were based on the assignment criteria of Rannala and Mountain [159] not assuming genetic equilibrium, and the re-sampling algorithm of Paetkau *et al*. [160] after 10.000 simulations and a threshold score of 5%. We applied IMMANC v.5.0 [159], with 10.000 iterations to verify F_0 _estimates and to assess the power of our dataset.

Population patterns of migration over the last generation within lakes and spawning sites were assessed in BIMR. The method implements estimation of inbreeding coefficients (F) to allow for departure from HW, and assumes sampling after reproduction, but before migration. Information used is gametic disequilibrium, and estimates are calculated using a Bayesian approach and MCMC technique, including 95% highest posterior density intervals (HPDI). Burn-in and sample size of 10^5^, thinning 50, F-model and default values of pilot runs, priors and incremental values were used, reporting mode estimates from the run with lowest total deviance and acceptance rate of 25 - 45% after 10 replicates to ensure convergence [44,100,147]. Bayesian assignment tests were then performed on individual level among lakes and among all sampled spawning sites in GENECLASS (with above settings). The random mid-lake sample was similarly assigned to populations, to test for temporal continuous distribution.

Patterns of recent admixed ancestry were finally evaluated in BIMR to assess hybrid presence within spawning sites with above settings on a population level. Hybrid ancestry was only considered on population level, including HPDI estimates, as hybrid detection under admixture may be hampered by restricted power. IMMANC was used to assess F1 ancestral origin on an individual level. Multiplicity correction procedure of Benjamini and Hochberg was used, balancing the risk of Type 1 and Type 2 errors at α = 5% [FDR correction; 45].

Forward modeling of population differentiation was performed with EASYPOP v.2.0.1. [161] to evaluate if genetic drift could cause observed differentiation patterns alone, and for power evaluation. Simulations were performed with variation in gene flow (0.01 - 0.99), and *N_e _*(30 - 90), for 5 populations in 20 generations following panmixia (*m *= 1.0) with maximum variation and 15 allelic states, assuming random mating, equal sex ratio and an island migration model. Observed pairwise population differentiation from our sample was compared with matrix scenarios of migration and *N_e _*indicating similar F_ST _estimates (above) from simulations. Ten independent replicates were obtained for the most likely model to address simulation variation. Similar backward modeling was run in SIMCOAL2 [162] to test the likelihood of the drift model.

## Authors' contributions

All authors; JW, JH and KH conceived the study and participated in the project design. JW conducted the field and laboratory work, and did the genetic analyses. All authors wrote and approved the final manuscript.

## Supplementary Material

Additional file 1 **Summary statistics**. Sample sites, number of analyzed individuals (n), number of amplified individuals (N), number of alleles (N_all_), expected and observed (direct count) heterozygosity (H_e _and H_o_). Significant departures from HW (P < 0.05) within sites after FDR correction are marked *. Click here for file

Additional file 2 **Coalescent simulated population differentiation**. Backward simulations in SIMCOAL2 supporting the findings of genetic drift as a contemporary driver for observed site fidelity. Click here for file
