# Supplementary material for: Population connectivity: dam migration mitigations and contemporary site fidelity in arctic char
Source: BMC Evol Biol. 2011 Jul 14;11:207. doi: 10.1186/1471-2148-11-207 (PMC3161007; doi:10.1186/1471-2148-11-207)
Supplement: Additional file 2 — Coalescent simulated population differentiation. Backward simulations in SIMCOAL2 supporting the findings of genetic drift as a contemporary driver for observed site fidelity. [file 1471-2148-11-207-S2.DOC]

Genetic drift scenarios simulated with a coalescent backward approach in SIMCOAL2, assuming a founder population of *N_e_* = 300 splitting into five populations of *N_e_* = 60 and constant size, 20 generations ago. We simulated independent chromosomes and a mutation rate of 10^-4^ in an IAM model constrained to 15 alleles. The right column states the proportion of pairwise comparisons that is significant after correction for multiple tests. Ten independent simulations of three migration rates (m = 0.05; 0.1; 0.2) revealing comparable F_ST_’s with observed values are given below.

| **Migration** | **Mean pairwise F_ST_ (θ)** | **± SD** | **P** | **% P < 0.05** | **% Sign. FDR** |
| --- | --- | --- | --- | --- | --- |
| 0,01 | 0,0675 | 0,0106 | < 0,0001 | 100 | 100 |
| 0,05 ^(a)^ | 0,0126 | 0,0038 | < 0,0113 | 100 | 100 |
| 0,1 ^(b)^ | 0,0048 | 0,0026 | < 0,3902 | 50 | 27 |
| 0,2 ^(c)^ | 0,0021 | 0,0024 | < 0,8077 | 18 | 6 |
| 0,3 | 0,0030 | 0,0032 | < 0.9219 | 30 | 10 |
| 0,4 | 0,0008 | 0,0019 | < 0.9824 | 10 | 0 |
| 0,5 | 0,0007 | 0,0011 | < 0.9727 | 0 | 0 |
| 0,99 | 0,0020 | 0,0020 | < 0.9121 | 0 | 0 |
|  |  | * a,b,c; mean over 10 simulations (below) | | | |
